# Maternal characteristics during pregnancy and risk factors for positive HIV RNA at delivery: a single-cohort observational study (Brescia, Northern Italy)

**DOI:** 10.1186/1471-2458-11-124

**Published:** 2011-02-21

**Authors:** Ilaria Izzo, Maria A Forleo, Salvatore Casari, Eugenia Quiros-Roldan, Michele Magoni, Giampiero Carosi, Carlo Torti

**Affiliations:** 1 Institute of Infectious and Tropical Disease, University of Brescia, Brescia, Italy; 2 Chair of Hygiene and Epidemiology, University of Brescia, Brescia, Italy

## Abstract

**Background:**

Detectable HIV RNA in mothers at delivery is an important risk factor for HIV transmission to newborns. Our hypothesis was that, in migrant women, the risk of detectable HIV RNA at delivery is greater owing to late HIV diagnosis. Therefore, we examined pregnant women by regional provenance and measured variables that could be associated with detectable HIV RNA at delivery.

**Methods:**

A observational retrospective study was conducted from January 1999 to May 2008. Univariate and multivariable regression analyses (generalized linear models) were used, with detectable HIV RNA at delivery as dependent variable.

**Results:**

The overall population comprised 154 women (46.8% migrants). Presentation was later in migrant women than Italians, as assessed by CD4-T-cell count at first contact (mean 417/mm^3 ^versus 545/mm^3^, respectively; p = 0.003). Likewise, HIV diagnosis was made before pregnancy and HAART was already prescribed at the time of pregnancy in more Italians (91% and 75%, respectively) than migrants (61% and 42.8%, respectively). A subgroup of women with available HIV RNA close to term (i.e., ≤30 days before labour) was studied for risk factors of detectable HIV RNA (≥50 copies/ml) at delivery. Among 93 women, 25 (26.9%) had detectable HIV RNA. A trend toward an association between non-Italian nationality and detectable HIV RNA at delivery was demonstrated by univariate analysis (relative risk, RR = 1.86; p = 0.099). However, by multivariable regression analysis, the following factors appeared to be more important: lack of stable (i.e., ≥14 days) antiretroviral therapy at the time of HIV RNA testing (RR = 4.3; p < 0.0001), and higher CD4+ T-cell count at pregnancy (per 50/mm^3^, RR = 0.94; p = 0.038).

**Conclusions:**

These results reinforce the importance of extensive screening for HIV infection, earlier initiation of antiretroviral therapy and stricter monitoring of pregnant women to reduce the risk of detectable HIV RNA at delivery. Public health interventions should be particularly targeted to migrant women who are frequently unaware of their HIV status at the time of pregnancy.

## Background

UNAIDS estimated that 31.3 million patients lived with HIV infection worldwide in 2008; among them, 15.7 million were women [1]. Before the introduction of combination antiretroviral therapy (cART), about 25% of infants born to HIV-infected women became HIV infected, but this risk was reduced by cART [1]. As a consequence, many women desire to have children despite HIV infection, while others become pregnant unaware that they are infected with HIV. For instance, more than 6,000 women living with HIV become pregnant in the United States every year [2].

Detectable HIV RNA at delivery is the strongest predictor of mother-to-child HIV transmission [3]. Katz et al. [4] examined the association between cART, demographic factors and detectable HIV-RNA at delivery. Alarmingly, 32% of the women displayed detectable HIV RNA at delivery, which was significantly associated with lower CD4+ counts and higher HIV RNA during pregnancy, but not type of cART [4]. A recent study of the European Collaborative Cohort highlighted a group of pregnant women with detectable HIV RNA at delivery notwithstanding cART, suggesting the importance of strict monitoring and support for HIV-infected pregnant women [5].

In Italy, characteristics of HIV-infected pregnant women and possible determinants of positive HIV RNA at delivery have been poorly studied [6]. In particular, maternal characteristics of migrants in comparison to autochthonous populations and the possible impact of migration on the risk of positive HIV RNA at delivery have not yet been investigated. We hypothesized that HIV infection is diagnosed in migrants later than in Italians, with consequences for the risk of detectable HIV RNA at delivery. First, we compared maternal characteristics of Italian and non-Italian women to determine whether there were any differences in terms of timing of presentation and cART initiation; second, we selected the women for whom HIV RNA close to term was available to explore possible risk factors for detectable HIV RNA at delivery, including patient nationality.

## Methods

### Characterisation of the overall population by geographical origin and management of pregnant women and newborns

A retrospective study was conducted on all pregnant women attending a large outpatient HIV clinic in Brescia (northern Italy) from January 1999 to July 2008. Pregnant women were initially referred to our clinic by general practitioners, obstetric services and services for sexually transmitted diseases at the time of HIV diagnosis, or were already on follow-up when they were found to be pregnant. On the basis of Italian guidelines for antiretroviral therapy available at the time of the study [7], the women were followed during pregnancy with examinations and clinical examinations every three months, using an integrated approach (HIV disease management and obstetric evaluation). Complete blood tests (including CD4+ T-cell count and HIV RNA) were performed, and the women were screened for latent tuberculosis infection, TORCH (*T. gondii*, others, Rubella, Cytomegalovirus, HIV) and malaria, if clinically indicated. HIV RNA was determined by branched DNA quantification (Bayer bDNA test, Versant HIV-1 RNA 3.0 assay [bDNA], Bayer Health Care). Antiretroviral treatment was commenced as soon as possible if indicated by the mothers' conditions [7]; otherwise, cART was initiated as soon as the mothers entered the 2^nd ^trimester of pregnancy. Italian guidelines edited in 2005 recommended beginning antiretroviral therapy at CD4+ T-cell count ≤350 cells/mm^3^; treatment at 350 < CD4+ < 500 cells/mm^3 ^was only suggested if HIV RNA was >100,000 copies/ml [7]. HIV-infected women were delivered by caesarean section, and zidovudine was infused as prophylaxis during delivery. Breastfeeding was discouraged.

The children were followed at the Paediatric Clinic of the local hospital (Spedali Civili di Brescia) with clinical evaluation and complete blood tests including HIV antibody test (ELISA) and HIV RNA on day 0, week 4, week 8, week 24, week 48 and week 96. Children were considered to be negative for HIV infection if HIV RNA and HIV antibody tests were negative at week 48.

This study was approved by the Ethical Committee of the Spedali Civili di Brescia. The patients signed the informed consent for participation to this study.

### Factors associated with detectable HIV RNA at delivery (subgroup of mothers with available HIV RNA close to term)

For this analysis, we considered only patients with available HIV RNA measured close to term (i.e., ≤30 days before delivery). Log-normal generalized linear regression models were used to assess factors associated with the outcome, defined as detectable HIV RNA (i.e., ≥50 copies/ml) close to term. Variables were selected on the basis of plausibility of their association with the outcome and epidemiological relevance, and to avoid possible biases. For example, CD4+ T-cell counts at delivery were not included, since antiretroviral therapy and time of initiation were likely to influence these values and HIV RNA at the same time. Therefore, the following variables were selected: geographical provenance (Italy versus other countries), age, calendar year at pregnancy, CD4+ T-cell count at pregnancy, and presence/type of cART at the time of HIV RNA testing (no therapy versus two antiretroviral drugs versus at least three drugs in combination, i.e., HAART regimens). Therapy was considered to be present if it was initiated at least 14 days before HIV RNA testing.

Since the 9 patients who were not prescribed cART at pregnancy were the same who were not on cART close to term, in the multivariable model we used a single dichotomy variable (no cART versus any cART close to term). Moreover, since all these 9 patients were non-Italians, there was an effect modification between country of origin and treatment exposure. For this reason, nationality was excluded from the main multivariable model. Lastly, in order to test whether nationality was correlated with detectable HIV RNA close to term, the 9 patients without treatment were excluded from a multivariable model that included nationality as variable.

Risk ratios (RR) with corresponding 95% confidence intervals were used to describe the strength of the associations.

### Additional definitions and statistical notes

The time of diagnosis of HIV infection was at the first positive HIV confirmatory test (western blotting), and the CD4+ T-cell count and HIV RNA at diagnosis were the first values obtained after HIV diagnosis. The time of pregnancy diagnosis was considered to be the last menstrual period, and CD4+ T-cell count and HIV RNA at pregnancy were the first values obtained after pregnancy diagnosis. Lastly, cART at delivery was the regimen at the last blood test before delivery.

The chi square test and score test for trend were used when appropriate. All statistical tests were two sided. P-values <0.05 were considered to be significant. Data were collected in an electronic chart used in our Centre (NetCare, version 1.05.11 sp 26) and double-checked for consistency and completeness with paper charts. Statistical analyses were performed using STATA software (Stata Statistical Software release 9.2, 2007; Stata Corporation, College Station, Texas).

## Results

### Characteristics of the population by geographical origin

One hundred and fifty-four pregnant women were included in the study (table 1). Most of the patients (46.7%) came from Italy or from Africa (42.4%), the remainder from Eastern Europe (6.3%) or from different countries (4.5%). The non-Italian patients had acquired HIV through heterosexual intercourse more frequently (p < 0.0001), and had received HIV diagnosis later (p < 0.0001) and during pregnancy (p < 0.0001), than the Italians. Two Italian women were infected vertically. Moreover, non-Italians had more previous pregnancies (p = 0.078), and presented with lower CD4+ T-cell count (p = 0.003), than Italians. Conversely, Italians were prescribed cART earlier than the non-Italians (p = 0.006).

**Table 1:** Characteristics of the cohort (overall and sub-study population)

	Pregnant women (all)				Pregnant women (HIV RNA available within 30 days before delivery)			
**Characteristic**	**N**	**Italians**	**Non-Italians**	**p**	**N**	**Italians**	**Non-Italians**	**p**

Age (mean years, SD)	154	33.7 (6.2)	29.8 (4.9)	<0.0001	93	32.8 (6)	30 (4.4)	0.004

Risk factor for HIV infection (N, %)	154				93			
Heterosexual intercourse		39 (54.2)	69 (84.1)			22 (56.4)	46 (85.2)	
IVDU		29 (40.2)	2 (2.4)			13 (33.3)	0	
Mother-to-child		2 (2.8)	0			2 (5.1)	0	
Parenteral		0	3 (3.7)			0	3 (5.6)	
Unknown		2 (2.8)	8 (9.8)	<0.0001		2 (5.1)	5 (9.3)	<0.0001

Calendar year at HIV diagnosis (N, %)	144				86			
≤1990		16 (23.9)	2 (2.6)			9 (25.7)	1 (2)	
1991-1995		19 (28.4)	1 (1.3)			10 (28.6)	1 (2)	
1996-2000		7 (10.4)	15 (19.5)			3 (8.6)	7 (13.7)	
2001-2005		20 (29.8)	42 (54.5)			10 (28.6)	28 (54.9)	
≥2006		5 (7.5)	17 (22.1)	<0.0001		3 (8.6)	14 (27.4)	<0.0001

Calendar year at pregnancy (N, %)	154				93			
1999		0	1 (1.2)			0	0	
2000		0	0			0	0	
2001		0	1 (1.2)			0	0	
2002		1 (1.4)	2 (2.4)			1 (2.6)	2 (3.7)	
2003		9 (12.5)	6 (7.3)			2 (5.1)	2 (3.7)	
2004		12 (16.7)	12 (14.6)			8 (20.5)	5 (9.3)	
2005		15 (20.9)	14 (17.1)			7 (18)	11 (20.4)	
2006		14 (19.4)	18 (22)			8 (20.5)	14 (26.9)	
2007		15 (20.8)	21 (25.6)			8 (20.5)	15 (27.8)	
2008 (January-July)		6 (8.3)	7 (8.6)	0.865		5 (12.9)	5 (9.3)	0.764

Previous pregnancies (mean, SD)	121	0.9 (1.1)	1.5 (1.7)	0.078	72	0.8 (0.7)	1.5 (1.5)	0.102

HIV diagnosis related to this pregnancy (N, %)	144				86			
Previous		61 (91.0)	47 (61.0)			32 (91.4)	29 (56.9)	
During pregnancy		6 (9.0)	30 (39.0)	<0.0001		3 (8.6)	22 (43.1)	0.001

Pregnancy outcome (N, %)	154				93			
Delivery		55 (76.4)	70 (85.4)			39 (100)	54 (100)	
Voluntary abortion		9 (12.5)	4 (4.9)					
Spontaneous abortion		5 (6.9)	7 (8.5)					
Therapeutic abortion		1 (1.4)	0					
Unknown		2 (2.8)	1 (1.2)	0.445				N.A.

CD4+/mm^3 ^at first contact (mean, SD)	154	545.6 (281.5)	417.5 (256.8)	0.003	93	495.1 (273.1)	398.7 (232.9)	0.070

CD4+/mm^3 ^at first contact (N, %)	154				93			
<200		2 (2.8)	12 (14.7)			2 (5.1)	9 (16.7)	
200-349		20 (27.8)	32 (39)			13 (33.3)	21 (38.9)	
350-499		18 (25)	16 (19.5)			10 (25.7)	11 (20.4)	
≥500		32 (44.4)	22 (26.8)	0.010		14 (35.9)	13 (24)	0.250

CD4+/mm^3 ^at pregnancy (mean, SD)	151	485.6 (247.3)	429.6 (252.9)	0.171	93	488 (222.1)	411 (217.2)	0.095

CD4+/mm^3 ^at pregnancy (N, %)	151				93			
<200		6 (8.3)	12 (15.2)			2 (5.1)	9 (17)	
200-349		19 (26.4)	27 (34.2)			12 (30.8)	18 (33.3)	
350-499		16 (22.2)	13 (16.4)			6 (15.4)	9 (16.7)	
≥500		31 (43.1)	27 (34.2)	0.300		19 (48.7)	18 (33.3)	0.265

Timing of antiretroviral therapy initiation (N, %)	125				93			
Before pregnancy		41 (74.5)	30 (42.8)			28 (71.8)	23 (42.6)	
1^st ^trimester		4 (7.3)	7 (10)			3 (7.7)	7 (13)	
2^nd ^trimester		8 (14.5)	19 (27.1)			7(18)	12 (22.2)	
3^rd ^trimester		1 (1.8)	5 (7.1)			1 (2.6)	4 (7.4)	
No therapy		1 (1.8)	9 (12.9)	0.006		0	8 (14.8)	0.026

Type of antiretroviral therapy at delivery; N (%)	125				93			
2 NRTIs		2 (3.6)	4 (5.7)			1 (2.6)	3 (5.6)	
3 NRTIs		1 (1.8)	0			1 (2.6)	1 (1.6)	
2 NRTIs + NNRTI		18 (32.7)	23 (32.9)			12 (30.8)	15 (27.8)	
2 NRTIs + PI		30 (54.5)	27 (38.6)			24 (61.5)	24 (44.4)	
Other regimens		4 (7.2)	3 (4.2)			1 (2.6)	1 (1.6)	
No cART		0	13 (8.6)	0.065		0	10 (18.5)	0.170

Type of antiretroviral therapy at delivery; N (%)	125				93			
No cART		0	13 (18.6)			0	10 (18.5)	
2 drugs		3 (5.4)	6 (8.6)			2 (5.1)	4 (7.4)	
≥3 drugs		52 (94.6)	51 (72.8)			37 (94.9)	40 (74.1)	0.014

N: the number of data available for the characteristics considered. IDVU: intravenous drug users; SD: standard deviation; NRTIs: nucleoside/nucleotide reverse transcriptase inhibitors; NNRTIs: non nucleoside/nucleotide reverse transcriptase inhibitors; PIs: protease inhibitors; cART: combination antiretroviral therapy; N.A.: not applicable.

**Figure 1:**
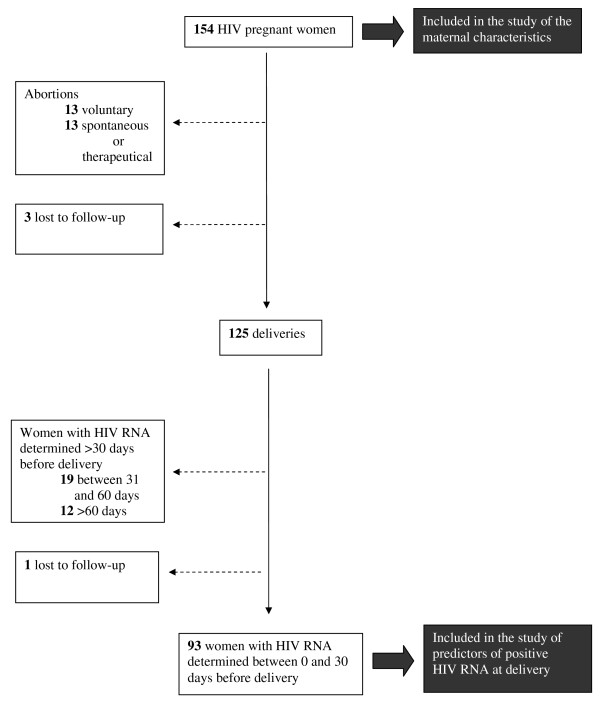
**Flow chart of the patients included in the study**.

Figure 1 depicts the flow of patients. Thirteen women had voluntary interruptions of pregnancy, 12 had spontaneous abortions, one had therapeutic abortion, and three were lost to follow-up soon after the first contact with the Centre. Therefore 125 women delivered. None of the children acquired HIV infection in this cohort.

### Factors associated with detectable HIV RNA at delivery

Among the 125 women who delivered, 93 had HIV RNA determination close to term (i.e., ≤ 30 days before delivery). Characteristics of these 93 women are illustrated in table 1. Moreover, CD4+ T-cell counts at delivery were slightly lower among non-Italians (mean: 464.7/mm^3^, SD 228.6/mm^3^) than Italians (mean: 582.1/mm^3^, SD 237.7/mm^3^) in the subset of patients with available HIV RNA determination close to term (p = 0.018). Significant differences were found between the 93 women with HIV RNA determination close to term and the remaining 61 women who lacked HIV RNA for CD4+ T-cell count at first contact (mean: 439.1 cell/mm^3^,SD 253.7/mm^3 ^*versus *535.7 cell/mm^3^, SD 298.2/mm^3^; p = 0.032) and initiation of cART before pregnancy (80.3% versus 54.8%, p = 0.014). No significant differences were found for the other variables between the two groups.

Factors considered for association with detectable HIV RNA at delivery and linear regression analysis are shown in table 2. Among the 93 women with available HIV RNA at delivery, 32/39 (82.0%) of the Italians versus 36/54 (66.7%) of the non-Italians had undetectable HIV RNA at delivery. Therefore, by univariate linear regression analysis, non-Italians had RR = 1.86 for detectable HIV RNA at delivery with respect to the Italians (p = 0.099). Age and calendar year were not statistically associated with the outcome.

**Table 2:** Univariate and multivariable regression analyses

Patient characteristics N = 93			Risk ratio for detectable HIV-RNA at delivery (95% confidence interval); p-value	
	**Patients with undetectable HIV-RNA at delivery N = 68**	**Patients with detectable HIV-RNA at delivery N = 25**	**Univariate analyses**	**Multivariable regression analyses using generalized linear models**

Nationality (N, %)				
Italian	32/39 (82.1%)	7/39 (17.9%)	Ref.	Excluded due to collinearity with cART
Non-Italian	36/54 (66.7%)	18/54 (33.3%)	1.86 (0.86- 4.01); p = 0.099	

Age (mean years, SD)	31.5 (5.5)	30.5 (4.99)	For each year 0.98 (0.93-1.04); p = 0.5	For each year 0.99 (0.94-1.05); p = 0.9

Calendar year at pregnancy (mean, SD)	2005.8 (1.6)	2005.6 (1.3)	For each year 0.93 (0.76-1.15); p = 0.7	For each year 0.96 (0.85-1.10); p = 0.6

cART at HIV RNA test (N, %)				
0 drugs	1/9 (11%)	8/9 (89%)	4.4 (2.7-7.1); p < 0.0001	Dichotomy variable (no cART - versus- any cART)
2 drugs	6/6 (100%)	0/6 (0%)		
≥3 drugs	61/78 (78%)	17/78 (22%)	Ref. 2 or ≥3 drugs (no differences among the 2)	4.4 (2.7-7.2) P < 0.0001
	
cART relative to pregnancy (N, %)				
before 2^nd ^trimester	49/60 (82%)	11/60 (18%)	Ref. any periods (no differences among the 3)	
2^nd ^trimester	14/19 (74%)	5/19 (26%)		
3^rd ^trimester	4/5 (80%)	1/5 (20%)		
No therapy	1/9 (11%)	8/9 (89%)	4.4 (2.7-7.1); P < 0.0001	

CD4+/mm^3 ^at pregnancy (mean, SD)	468 (223)	376 (204)	For each 50 CD4+/mm^3 ^0.91 (0.84-0.99); P = 0.037	For each 50 CD4+/mm^3 ^0.94 (0.89-0.99); P = 0.032

Analyses were on patients with plasma viral load measured at the time of delivery (within 30 days before delivery). cART: combination antiretroviral therapy; Ref.: reference category.

Among nine patients who were not on cART for ≥14 days at the time of HIV RNA testing, only one (11%) had undetectable HIV RNA. By contrast, among 78 patients who were on ≥3 antiretroviral drugs, 61 (78%) had undetectable HIV RNA and this percentage was not statistically different from the percentage of patients with undetectable HIV RNA among those who were prescribed two antiretroviral drugs (6/6 = 100%). Therefore, by univariate analysis, the RR of detectable HIV RNA was 4.4 (p < 0.0001) for lack of cART versus any cART at the time of HIV RNA testing. Similar results were obtained for presence/timing of initiation of cART, resulting in an identical RR of detectable HIV RNA for lack of cART versus any cART initiated before or during pregnancy.

Lastly, increasing CD4+ T-cell count at pregnancy appeared to reduce the risk of detectable HIV RNA at delivery by univariate analysis (RR = 0.91 per 50 cells/mm^3^; p = 0.037). All continuous variables included in the regression model were also studied as categorical variables to confirm the linear relationship; in particular, upper classes of CD4+ T-cell counts (200-349; 350-499; >500/mm^3^) were associated with a lower risk of detectable HIV RNA at delivery with respect to CD4+ <200/mm^3 ^(data not shown).

Multivariable analysis confirmed that the risk of detectable HIV-RNA at delivery was increased by lack of cART, while higher CD4+ T-cell count at the beginning of pregnancy appeared to be protective; by contrast, age and calendar year did not appear to be associated with HIV-RNA at delivery (see Table 2). After excluding from the analysis the 9 patients who were not receiving any cART, we found that nationality was not independently associated with the risk of detectable HIV RNA close to term (RR = 0.91; p = 0.8). Also, in this model, results for the other variables did not change significantly. In particular, the protective role of CD4+ T-cell count at pregnancy was confirmed (risk ratio = 0.87 per 50 cells/mm^3^; p = 0.049).

## Discussion

Increasing numbers of HIV-infected women are becoming pregnant or are planning a pregnancy owing to the widespread use of cART and a decrease in HIV-related mortality and morbidity in developed countries [8]. In our HIV cohort we included 154 pregnant women from January 1999 to July 2008. More than half of them were migrants (especially from Africa), reflecting the burden of immigration in Italy in recent years [6,9]. Importantly, more than one quarter of the migrants were diagnosed with HIV at the time of pregnancy, underlining the fact that HIV would have been diagnosed before pregnancy if the screening policy were more extensively implemented, thus allowing cART to be initiated earlier, in line with current recommendations [2]. Indeed, current Italian guidelines suggest treatment irrespective of CD4+ count in case of pregnancy. At the same time, this finding indicates that pregnancy offers a unique opportunity for HIV screening, especially in high-risk populations of migrant women.

Likewise, CD4+ T-cell counts at the first contact with our clinic were significantly lower among non-Italian women than Italians, reflecting more advanced stages of HIV infection and a later HIV diagnosis in the former. Furthermore, CD4+ T-cell counts at delivery were lower in the subset of non-Italian patients with available HIV RNA at delivery. It has already been demonstrated that lower CD4+ T-cell count at delivery is a risk factor for mother-to-child transmission of HIV [10]. Once again, these considerations reinforce the need for earlier diagnosis and treatment of HIV infection, particularly in migrant women.

It is important to note that, in about one quarter (31/125) of the women who delivered, HIV RNA was not measured in the 30 days before delivery, making it impossible to identify the risk of transmission to the newborn, suggesting appropriate interventions (e.g., treatment change or intensification, therapeutic drug monitoring to confirm low adherence and/or poor drug bioavailability, modification of drug dosage or counselling to improve adherence). The Italian guidelines for antiretroviral therapy edited in 2005 [7] suggested similar schedules of follow-up for pregnant women and for the other patients (every 2-3 months). The lack of HIV RNA determination close to term was due to the fact that 26/61 (42.6%) women had abortions and 3/61 were lost to follow-up. Furthermore, the lack of HIV RNA determination in the remaining 32/61 (52.5%) women was due to compliance with the previous guidelines, which suggested an infrequent schedule of monitoring rather than lack of patient adherence to the scheduled follow-up. Although none of the mothers transmitted the infection, such infrequent follow-up constituted a potential risk just because HIV RNA was not determined, so the risk was not identified and appropriately addressed. In view these considerations, the most recent Italian guidelines for antiretroviral treatment [11] has suggested that the follow-up of pregnant women should be stricter, at least in those who have started or modified cART during pregnancy. Moreover, further HIV RNA determinations are now recommended between the 34^th ^and the 36^th ^weeks of gestation [11]. Our findings support these recommendations.

In 25/93 (26.9%) women in whom HIV RNA was tested close to term, detectable HIV RNA values were found. Percentages of detectable HIV RNA at delivery were even greater in previous studies [4,5], highlighting the importance of studies on risk factors for detectable HIV RNA at delivery to optimize the clinical management of HIV in pregnant women.

In the subgroup of 93 women with available HIV RNA, a statistically significant association was found between risk of detectable HIV RNA at delivery and lower CD4+ T-cell count at pregnancy onset. This finding is not new since it was previously demonstrated by Katz et al. [4], indicating that initiation of cART when the immune system is less compromised could help reduce HIV RNA to undetectable levels at delivery. Interestingly, type of cART (dual or triple therapy) did not appear to influence the risk of detectable HIV RNA at delivery, while any cART had a significant protective effect, even though it was initiated during later stages of pregnancy. This finding is reassuring because it suggests that cART has a protective effect even though it is initiated late in pregnancy, but only in cases where earlier treatment is not feasible owing to late diagnosis of HIV infection. Indeed, our results must be interpreted with caution and must not be used to conclude that cART can be initiated later and with suboptimal regimens. In fact, dual therapy is not recommended [7]. Moreover, our sample size was extremely small for some categories (only six patients were treated with dual cART and only five started cART in the third trimester). Clearly, a larger number of patients might have altered the results.

Our study was the first to investigate the potential effect of patient nationality on the risk of detectable HIV RNA at delivery. There was only a trend toward a statistically significant effect of non-Italian nationality by univariate analysis, and this disappeared on multivariable analysis. Once again, the small number of patients could have influenced the results, but it is possible that behavioural factors associated with nationality (i.e., late HIV diagnosis and late initiation of cART) or other factors not captured in this analysis (e.g., low patient adherence or unmeasured characteristics of the virus such as HIV drug resistance or viral subtype [12]) were more important than nationality "per se". Therefore our results suggest that early diagnosis and treatment, though prioritized in the migrants, should be extended to the overall population. A screening campaign in the general population and adherence to the recommendation to test pregnant women for HIV are mandatory.

This study has several limitations that need to be recognised. First, it was a retrospective analysis, so several items of information were not recorded. In particular, because they were not available for the earliest calendar years, HIV drug resistance testing, viral subtypes or therapeutic drug monitoring results were not considered. Second, as previously discussed, the sample size was small. Third, HIV RNA was available at delivery for only 93 women. Therefore, there should be further investigation of maternal characteristics and pregnancy outcomes in migrant and autochthonous women using prospective cohorts with completed data and larger sample size.

## Conclusions

Overall our results reinforce the recommendation to start HAART as soon as possible in pregnant women. Clearly, this is only achievable by testing for HIV as soon as possible. Migrant patients appeared to be a vulnerable population because they were more frequently unaware of their HIV status at the time of pregnancy than Italians. Since there is no nationwide legislation that imposes HIV-testing in pregnant women in Italy, our results have important public health implications. Moreover, they indicate the need for a strict virological and clinical follow-up of pregnant women to detect amendable causes of positive HIV RNA at delivery to minimize the risk of HIV transmission.

## Competing interests

CT and GC have received unrestricted educational grants (as speakers or for participation to conferences) from Abbott, Gilead, Merck, GSK, BMS, Schering Plough, Roche. The remaining authors declare no competing interests.

## Authors' contributions

CT, GC and II conceived of the study, participated in its design and helped to draft the manuscript. MAF, SC and EQ participated in design of the study and acquisition of data. MM participated in design of study and performed the statistical analysis. All authors read and approved the manuscript.

## Pre-publication history

The pre-publication history for this paper can be accessed here:

http://www.biomedcentral.com/1471-2458/11/124/prepub
